# 

**Published:** 1915-03-20

**Authors:** 


### Gifts and Bequests.

A legacy of £500, free of duty, to the West End Hospital, has been announced from the late Mr. W. Edgar Allen, an old subscriber.

The Chelsea Hospital for Women has received from the estate of the late Mr. Edward Baker a sum of £4,788 4s. 1d., of which £2,640 is for the rebuilding, and the remainder for the general fund.

Mr. William Edgar Allen, of Sheffield, has left about £150,000 in bequests to charities and employees. The Sheffield local charities are the chief recipients. The legacies also include £1,000 each to the Hospital for Consumption at Ventnor and the Mission to Lepers in India.

Among the latest contributions received at the Bank of England for King Edward's Hospital Fund for London are the following annual subscriptions:—Mr. Walter Morrison, £5,000; Mr. William Waldorf Astor, £1,000; Messrs. C. J. Hambro and Son, £250; Prudential Assurance Company (Limited), £250; and Mrs. Walter H. Burns, £200.

Mr. James Robinson, of Blackpool, wine and spirit merchant, in the event of the failure of certain trusts, has left bequests to the Preston Royal Infirmary, the Victoria Hospital, Blackpool, and a local mission. The testator also left £1,500 upon trust for his housekeeper for life, with remainder to the Victoria Hospital, Blackpool, together with £20 for each year of her service, and £1,500 upon trust for his sister for life, with remainder to the Preston Royal Infirmary. Subject to a few other bequests, he left the residue of his estate in equal shares to ten local charities.

### Order of St. John of Jerusalem.

The King has sanctioned the following promotions in and appointments to the Order of the Hospital of St. John of Jerusalem in England :—

*As Knights of Justice (from Knights of Grace).*—J. H. Stevenson, Col. F. W. Pixley.

*As Chaplain.*—The Bishop of Peterborough.

*As Knights of Grace.*—T. O. Fraser (from Esquire), Col. W. J. Will, M.B., A.M.S. (N.Z.), Col. Sir W. R. Lawrence, Bt., G.C.I.E., the Hon. Arthur Stanley, M.V.O., M.P., Lieut.-Col. F. Marsh, F.R.C.S., M.B., R.A.M.C. (T.F.), Col. Sir Claude M. MacDonald, P.C., G.C.M.G., G.C.V.O., K.C.B., the Hon. W. H. Goschen, J. Lumsden, M.D., Lord Norreys, Sir E. F. S. Flower, W. H. Winney (from Honorary Serving Brother), J. A. F. Aspinall, Lieut.-Col. E. J. King, F. S. E. Drury, Sir Henry Seymour King, K.C.I.E., Sir Mackenzie D. Chalmers, K.C.B., C.S.I., Sir W. Q. Ewart, Bt.

*As Ladies of Grace.*—Countess Grosvenor, Lady Bowater, Lady Pellatt, Mrs. R. McKenna, Miss J. L. Freshfield, Mrs. J. C. Lines (from Honorary Serving Sister), Miss F. E. Henderson, Lady Strathcona.

*As Esquire.*—P. G. Darvil-Smith (from Honorary Serving Brother).

### The First Certified Hospital Fire Brigade.

The following are the names of those who have won certificates at the Hospital St. Cross, Rugby, for proficiency in fire drill as a result of the examination lately held under the auspices of the National Fire Brigades' Union :—Matron : H. Florence Osborne. Nurses : Eleanor Evans, Violet Taylor, Amy Lester, Amy Butterworth, Joan Hollands, Elsie Secker, Gwendoline Cook, Violet Vaughan, Margaret Fry, Marion Crowley, Norah Holland, and Marjorie Campbell. Servants : Carrie Day, Edith Day, Elsie Davenport, Agnes Hirons, Florrie Day, and Norah Charlwood. It should be placed on record that the Hospital of St. Cross, Rugby, is the first hospital to be examined under the scheme adopted by the National Fire Brigades' Union. Particulars of the scheme can be obtained from Mr. Augustus Slater, secretary, 22 Northumberland Avenue, W.C.

### Medical Appointments.

Dr. J. W. Pugh has been appointed Registrar at the West End Hospital in place of Dr. W. H. Bailey, who has resigned. Dr. Pugh holds the qualifications of M.D. Lond., M.R.C.S., and L.S.A. For some time he has held a clinical assistantship at the hospital, and since the outbreak of the war has been seeing out-patients for the physicians both at the West End and at one of the chest hospitals

### Doctors' Wills.

Deputy-Surgeon-General George Alder Watson, aged eighty-three, of Hendre, Overton Park, Cheltenham, late of the Indian Medical Service, a Crimean and Indian Mutiny veteran, who also saw service in the Afghan War, 1878-9, has left unsettled property of the value of £64,303.

### Buildings Contemplated.

Bexhill.—The Local Government Board have held an inquiry into an application by the Town Council for sanction to borrow £6,500 for the erection of new isolation hospital buildings at Clinch Green.

Derby.—The Guardians have resolved to build a new infirmary at a cost of £35,000.

Gateside.—The Greenock and District Combination Hospital Board are considering a proposal to extend the Gateside Hospital at an estimated cost of £1,800.

Heathfield.—The Ayr Dean of Guild Court has passed the plans submitted by the Ayr Town Council for an addition to Heathfield Hospital, estimated to cost £1,850.

Hothfield.—The Kent County Council have authorised the Asylums and Mental Deficiency Act Committee to acquire land and to provide an institution for the mentally defective. The site of the proposed institution is between Charing and Hothfield.

Lancaster.—The Town Council have received sanction from the Local Government Board to borrow £1,000 for extending the isolation hospital.

Londonderry.—The Local Government Board have held an inquiry into a petition by the Port Sanitary Authority for sanction to a loan of £1,100 for building an intercepting hospital for the Port of Londonderry to receive patients suffering from cholera, yellow fever, or plague.

Manchester.—The South Manchester Board of Guardians invite tenders for the erection of a hospital block at Withington. Quantities may be obtained of Mr. F. H. Overmann, 49 King Street, Manchester.

Newbold.—The North Derbyshire Isolation Hospitals Committee have decided to build convalescent blocks for the isolation hospital at Morton, Mastin Moor, Langwith, and Newbold.

Ripon.—Tenders are invited for alterations to the Ripon Cottage Hospital. Application should be made o the architects, Messrs. Brown and Crosland, 6 Princes Square, Harrogate.

Wallsend.—The Town Council have decided to apply for a loan of £4,479 9s. 2d. for extending the hospital.

### The Book Market.

LIST OF NEW BOOKS RECEIVED FROM THE FOLLOWING PUBLISHERS :—

Edward Arnold : "Surgical After-Treatment." By Alan H. Todd, B.Sc., M.S. 4s. 6d. net.

J Bale, Sons and Danielsson, Ltd. : "The Inevitable Complement : the Care and After-Care of Consumptives." By Harold Vallow, M.D. 1s. 6d. net.

A. and C. Black : "Radiography, X-Ray Therapeutics, and Radium Therapy." By Robert Knox, M.D. (Edin.), M.R.C.S. 25s. net.

Constable and Co., Ltd., for the General Medical Council : "The Medical Register, 1915," "The Dentists' Register, 1915."

C. W. Daniel, Ltd. : "A Key to Health and Long Life." By F. W. D. Mitchell, I.S.O. 3s. 6d. net.

J. M. Dent and Sons, Ltd. : "A Plea for the Thorough and Unbiassed Investigation of Christian Science." By Chas. Herman Lea. 1s. net.

Morgan and Scott, Ltd. : "The Gospel of Healing." By the Rev. A. B. Simpson, D.D. 2s. net.

Sir Isaac Pitman and Sons, Ltd. : "English Medical Women." By A. H. Bennett. 3s. 6d.

RATES OF SUBSCRIPTION: Twelve months, 6/6; Six months, 4/-; Three months, 2/-, post free to any address in the United Kingdom. Abroad, Twelve months, 8/8; Six months, 5/-; Three months, 2/6, post free.—Address The Subscription Dept., "THE HOSPITAL," The Hospital Building, 28/29 Southampton Street, Strand, London, W.C.

